# m^6^A modification plays an integral role in mRNA stability and translation during pattern-triggered immunity

**DOI:** 10.1073/pnas.2411100121

**Published:** 2024-08-08

**Authors:** Tianyuan Chen, George H. Greene, Jonathan Motley, Musoki Mwimba, Guan-Zheng Luo, Guoyong Xu, Sargis Karapetyan, Yezi Xiang, Chang Liu, Chuan He, Xinnian Dong

**Affiliations:** ^a^HHMI, Duke University, Durham, NC 27708; ^b^Department of Biology, Duke University, Durham, NC 27708; ^c^HHMI, University of Chicago, Chicago, IL 60637; ^d^Department of Chemistry, University of Chicago, Chicago, IL 60637

**Keywords:** plant immunity, m^6^A, RNA decay, translation efficiency

## Abstract

Plants employ distinct mechanisms to respond to environmental changes. Modification of mRNA by *N*^ 6^-methyladenosine (m^6^A), known to affect the fate of mRNA, may be one such mechanism to reprogram mRNA processing and translatability upon stress. However, it is difficult to distinguish a direct role from a pleiotropic effect for this modification due to its prevalence in RNA. Through characterization of the transient knockdown-mutants of m^6^A writer components and mutants of specific m^6^A readers, we demonstrate the essential role that m^6^A plays in basal resistance and pattern-triggered immunity (PTI). A global m^6^A profiling of mock and PTI-induced *Arabidopsis* plants as well as formaldehyde fixation and cross-linking immunoprecipitation-sequencing of the m^6^A reader, EVOLUTIONARILY CONSERVED C-TERMINAL REGION2 (ECT2) showed that while dynamic changes in m^6^A modification and binding by ECT2 were detected upon PTI induction, most of the m^6^A sites and their association with ECT2 remained static. Interestingly, RNA degradation assay identified a dual role of m^6^A in stabilizing the overall transcriptome while facilitating rapid turnover of immune-induced mRNAs during PTI. Moreover, polysome profiling showed that m^6^A enhances immune-associated translation by binding to the ECT2/3/4 readers. We propose that m^6^A plays a positive role in plant immunity by destabilizing defense mRNAs while enhancing their translation efficiency to create a transient surge in the production of defense proteins.

## Significance

Pattern-triggered immunity (PTI) is a rapid, transient immune response in plants involving reprogramming the transcriptome and the translatome. Though *N*^6^-methyladenosine (m^6^A) modification has been well known to affect the fate of messenger RNA (mRNA), whether it plays a role in regulating plant immunity remained unclear. Our study demonstrates that m^6^A modification of mRNA is crucial for the induction of PTI in plants. Mutants of m^6^A “writer” components and “readers” showed compromised resistance to pathogens. Through multiomics analysis, we uncovered dual role of m^6^A during PTI in promoting rapid turnover of defense-related transcripts while enhancing their translation through association with different m^6^A readers to orchestrate a swift and effective defense response while minimizing penalty to plant growth.

*N*^6^-methyladenosine (m^6^A) is the most abundant modification in mRNA and has emerged as an important regulator of gene expression (1). In plants, this modification is catalyzed by the m^6^A methyltransferase complex (“m^6^A writer”) consisting of the catalytic subunits MTA (mRNA adenosine methylase, the ortholog of the mammalian METTL3) and MTB (METTL14) (2, 3), as well as the regulatory subunits FIP37 (FKBP12 INTERACTION PROTEIN 37KD, the ortholog of the mammalian WTAP), HAKAI, and VIRILIZER (4, 5). The m^6^A modification can be reversed through the activity of “eraser” proteins, such as the ALKBH (ALKB Homolog) family of demethylases (6). The presence of m^6^A modification in mRNA is then recognized and “interpreted” by “reader” proteins, such as the ECT (Evolutionarily Conserved C-Terminal region) family (7–9). The balance of this m^6^A system (i.e., activities of the writers, the erasers, and the readers) has been implicated in modulating cytosolic mRNA stability (10, 11). While FIP37-mediated m^6^A modification accelerates the degradation of certain development-related mRNAs in *Arabidopsis* (5), a more global monitoring of the m^6^A landscape revealed a stabilizing effect of MTA-mediated m^6^A modification (12). Consequently, under which circumstances m^6^A modification leads to mRNA degradation or stabilization in plants is unclear (13). This uncertainty could be due to the expanded ECT family in plants, which likely possess a broader range of functions than those found in mammals. For instance, the readers ECT2 and ECT3 are associated with the stabilization of m^6^A-modified mRNAs (9, 14, 15), whereas ECT1 contributes to RNA degradation (16). YTHDF m^6^A readers in mammals have also been implicated in the regulation of the translation efficiency of the modified mRNAs (17), while in plants, the role of m^6^A in regulating the translation efficiency of modified transcript remains underexplored (13).

Regulation of gene expression is essential for eukaryotes to respond to abiotic and biotic stresses, especially for plants, which are sessile organisms. Indeed, in plants, m^6^A modification has been shown to impact responses to salt, light stress, and chilling through regulation of mRNA stability or cytosolic availability for translation (12, 18, 19). However, the role of m^6^A in plant immunity has been ambiguous, because evidence for a positive as well as a negative role in basal resistance has been reported (16, 20, 21), indicating that a better distinction between a pleiotropic effect and a direct role of m^6^A modification in different immune responses is required.

In this study, we tested our hypothesis that alterations in mRNA fate during pattern-triggered immunity (PTI) may require dynamic changes in the balance of m^6^A deposition by writer proteins and interpretation by reader proteins. We focus on PTI because, as it is the first line of plant immune response, it depends on the detection of microbe-associated molecular patterns (MAMPs), and subsequent reprogramming of not only the plants’ transcriptome, but also the translatome (22, 23). We found that the m^6^A modification is required for both basal immunity and PTI. While the total abundance of the modification is unaltered in response to MAMP treatment, nascent modifications appear in the transcriptome after PTI induction. Interestingly, in mRNAs transcriptionally induced by PTI, the m^6^A writer complex promotes both increased decay and enhanced translational activity mediated by distinct ECT readers. Therefore, the outcome of m^6^A modification in PTI is not only dependent on deposition of the modification, but also distinct activities of the m^6^A reader proteins.

## Results

### m^6^A Modification Is Required for Plant Immunity.

**Fig. 1.**
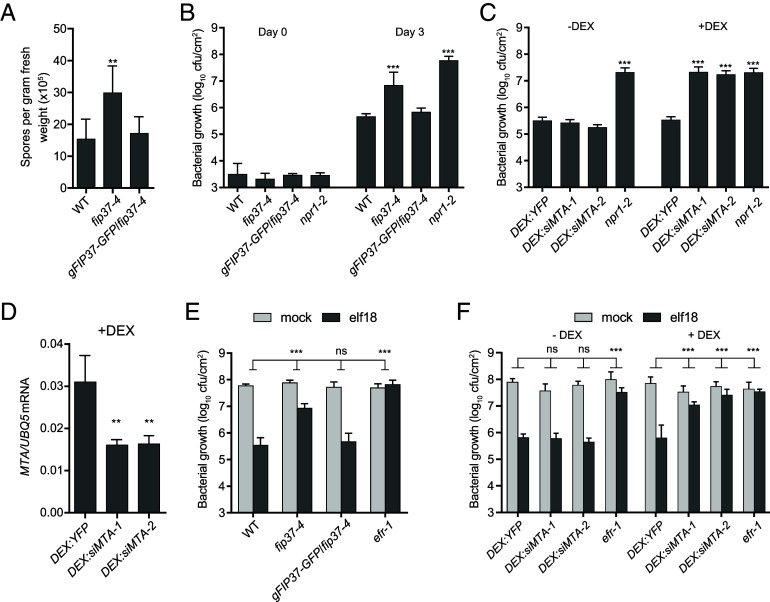
The m^6^A-deficient plants have compromised immune phenotypes. (*A*) Basal resistance to the oomycete pathogen *Hyaloperonospora arabidopsidis* Noco2 (*Hpa* Noco2). 12-d-old seedlings were sprayed with *Hpa* Noco2 spores (3 to 5 × 10^4^ spores/mL). Spores were collected and quantified 7 d after infection (n = 5). *gFIP37-GFP/fip37-4*, complementation line with the genomic *FIP37* promoter and coding sequence fused to GFP transformed in the *fip37-4* background. (*B* and *C*) Basal resistance to the bacterial pathogen *Pseudomonas syringae* pv *maculicola* ES4326 (*Psm* ES4326). Leaves from 3.5-wk-old plants were infiltrated with *Psm* ES4326 (OD_600nm_ = 0.0001). Bacterial growth was scored on Day 0 (n = 4) and Day 3 (n = 8). For the *DEX:siMTA* silencing lines (two independent transformants), plants were pre-treated with 50 µM Dexamethasone (+DEX) or H_2_O (−DEX) 1 d prior to infection. *npr1*, the *nonexpresser of* *pathogenesis-related 1* mutant known to have enhanced disease susceptibility. (*D*) The *MTA* transcript abundance in *DEX:siMTA* plants 24 h after DEX treatment compared to the *DEX:YFP* control. (*E* and *F*) elf18-induced resistance to bacteria. Leaves from 3.5-wk-old plants were infiltrated with 1 µM elf18 or mock (H_2_O). After 1 d, the same leaves were infiltrated with *Psm* ES4326 (OD_600nm_ = 0.001) and bacterial growth was scored 2 d later (n = 8). *DEX:siMTA* plants were sprayed with DEX 1 d prior to elf18 infiltration. All error bars represent 95% CI. Data (*A–D*) were analyzed by the Student’s *t* test. Two-way ANOVA with the Bonferroni post hoc test was performed for the comparison between mutants and WT (*E*) or DEX:YFP (*F*). **P < 0.01; ***P < 0.001; ns, not significant. Experiments were repeated at least twice with similar results.

To determine whether the m^6^A modification could affect plant defense against pathogens, we characterized the *fip37-4* mutant, a developmentally viable partial knockdown mutant of m^6^A modification (5), in response to pathogen challenge. We first examined the basal resistance of *fip37-4* plants to a natural oomycete pathogen of *Arabidopsis*, *Hyaloperonospora arabidopsidis* (*Hpa*) Noco2 (3-5 × 10^4^ spores/mL), and a bacterial pathogen, *Pseudomonas syringae* pv *maculicola* (*Psm*) ES4326 (OD_600nm_ = 0.0001). As a negative control for the bacterial infection experiment, a mutant with enhanced disease susceptibility, *nonexpresser of pathogenesis-related genes 1* (*npr1*), was included. These infection assays revealed significantly increased growth of *Hpa* Noco2 and *Psm* ES4326 in the *fip37-4* mutant compared to wild-type (WT) plants, and this enhanced susceptibility was rescued in the *gFIP37-GFP/fip37-4* transgenic complementation plants (5) (Fig. 1 *A* and *B*). Since the m^6^A modification is present in thousands of mRNAs even in the absence of pathogen challenge, and the transcriptome-wide m^6^A abundance is reduced in *fip37-4* (5), it is likely that the mutant has distinctly different basal transcriptome from that of the WT due to a high level of genetic interference both during development and at homeostasis. In order to more discretely observe the effects of m^6^A modification on plant immunity, we generated dexamethasone (DEX)-inducible silencing lines against the *MTA* (*DEX*:*siMTA*), the catalytic component of the m^6^A writer complex (12), to inhibit m^6^A modification only after the plants have developed normally and reached maturity. We found that without DEX treatment, the two independent *DEX:siMTA* lines showed bacterial growth at levels similar to that of the *DEX:YFP* control plants. However, after DEX treatment for 24 h, which reduced *MTA* mRNA levels to 50%, bacteria grew to higher levels, similar to *fip37-4* and *npr1* (Fig. 1 *C* and *D*). These results confirm the positive role of FIP37 and MTA in plant defense and suggest a general requirement for the m^6^A machinery in basal immunity.

To further distinguish a direct involvement of m^6^A modification in plant defense from a pleiotropic effect, we examined the responsiveness of the *fip37-4* mutant plants to MAMP by first infiltrating plants with 1 µM of elf18, an epitope of bacterial EF-Tu, followed by inoculation with *Psm* ES4326 (OD_600nm_ = 0.001 which is 10-fold higher than that used for detecting a defect in basal resistance), with the elf18 receptor mutant, *efr-1*, as the negative control. We found that the protection against *Psm* ES4326 conferred by the elf18-pretreatment was significantly compromised in the *fip37-4* mutant. Complementation of the mutant phenotype was achieved in the *gFIP37-GFP/fip37-4* line (Fig. 1*E*). Consistently, the two independent *DEX:siMTA* lines also showed a significant loss of elf18-mediated protection against *Psm* ES4326 infection after the DEX treatment (Fig. 1*F*), demonstrating that deposition of the m^6^A modification is needed for establishing PTI.

### PTI Induces Changes in m^6^A Modification.

Based on liquid chromatography–tandem mass spectrometry (LC-MS/MS) analysis, we found that in response to elf18 treatment, no discernable alteration in the total m^6^A abundance was detected in mRNA (*SI Appendix*, Fig. S1*A*). To further investigate the dynamics of m^6^A upon PTI induction, we performed global m^6^A-sequencing assays of mock- and elf18-treated WT plants. While m^6^A modifications were detected along the coding sequences (CDS) of mRNAs, there was a preference for the stop codon, consistent with previous reports (5, 12, 24), in both mock- and elf18-treated samples (*SI Appendix*, Fig. S1 *B* and *C*). The canonical m^6^A consensus, RRACH, was significantly overrepresented in the modified regions when compared to a randomized background (*SI Appendix*, Fig. S1*D*).

Despite the lack of an overall change in the total m^6^A abundance in response to elf18, we found 623 modifications in 518 mRNAs that were specific to the mock-treated plants, while 614 modifications in 459 mRNAs that were specific to the elf18-treated treatment, in addition to the 5008 shared modifications in 4098 transcripts (*SI Appendix*, Fig. S1*E* and Dataset S1). Among the 459 elf18-specific genes, 126 were found to be transcriptionally induced upon elf18 treatment, and the GO terms for the elf18-specific transcripts were enriched with “response to biotic stimulus,” such as immune regulators *EDS5*, *XLG3,* and *WRKY27*, suggesting that m^6^A may have a role in the newly synthesized defense-related transcripts during PTI (*SI Appendix*, Fig. S1*F*).

To examine whether specific *cis*-elements are involved in regulating the deposition of m^6^A in response to elf18, we searched for enriched sequences in both the m^6^A modified regions and the 150 nucleotides preceding and following the modification sites. Interestingly, clear consensus sequences were detected in the flanking regions, with a degree of overlap between mock and elf18-treated samples such as “GAAGAAGA” (*SI Appendix*, Fig. S1*G*). Therefore, condition-specific m^6^A modifications detected in our sequencing experiments likely result from a combination of transcript level changes and some specific *cis-*elements.

### m^6^A Readers ECT2/3/4 Positively Regulate PTI.

**Fig. 2.**
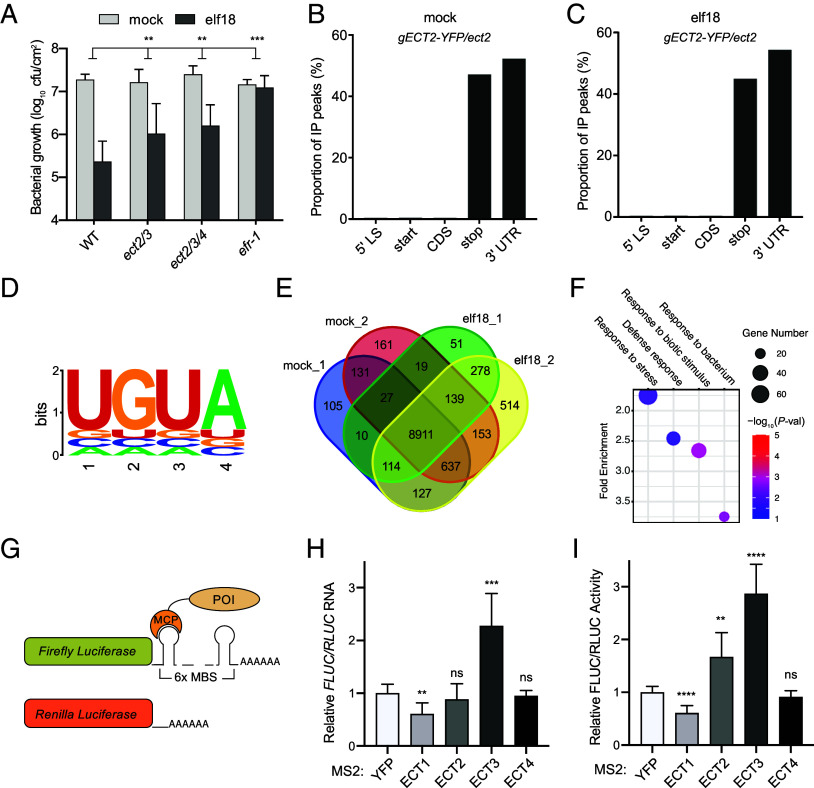
The m^6^A reader proteins ECT2/3/4 positively regulate PTI. (*A*) Resistance to bacterial infection induced by elf18. Leaves from 3.5-wk-old plants were infiltrated with 1 µM elf18 or mock 1 d prior to infection. The same leaves were infiltrated with *Psm* ES4326 (OD_600nm_ = 0.001) and bacterial growth was scored 2 d later (n = 8). Error bars represent 95% CI. (*B* and *C*) Distribution of enriched peaks in FA-CLIP-seq of ECT2 under mock (*B*) or elf18 (*C*) condition. *gECT2-YFP/ect2*, complementation line with the genomic *ECT2* promoter and coding sequence fused to YFP transformed in the *ect2* background. 5′ LS, 5′ leader sequence; start, start codon ± 50 nt; CDS, coding sequence; stop, stop codon ± 50 nt; 3′ UTR, 3′ untranslated region. (*D*) ECT2-binding consensus sequence detected in the center of enriched peaks. (*E*) Venn diagram of ECT2-bound transcripts after mock- or elf18-treatment. (*F*) Biological process Gene Ontology terms enriched in elf18-specific ECT2-bound transcripts. (*G*) Schematic diagram of the MS2-tethering system. POI, protein of interest; MCP, MS2 coat protein; MBS, MS2-binding sequences. (*H* and *I*) Effects of ECT proteins on the *FLUC* mRNA abundance (*H*) and translation (*I*). Individual ECT proteins were tethered to the 3′ UTR of the *FLUC* mRNA using the MS2 tethering system, and *RLUC* was coexpressed as the internal control. mRNA abundance and dual luciferase activity were quantified 2 d after *Agrobacteria* infiltration into *N. benthamiana* leaves (n = 3). Data were analyzed by two-way ANOVA (*A*) with the Bonferroni post hoc test and *t* test (*H* and *I*). **P < 0.01; ***P < 0.001; ****P < 0.0001; ns, not significant. Experiments were repeated at least twice with similar results.

After establishing the dynamics of m^6^A modification in the transcriptome upon immune induction, we next tested our hypothesis that binding of the m^6^A reader proteins to modified- mRNAs plays a role in the dynamic posttranscriptional regulation of gene expression during PTI. We characterized the response of mutants of the putative m^6^A readers, which are proteins containing a known m^6^A-binding YT521-B homology (YTH) domain. In *Arabidopsis*, 11 proteins have such YTH domains in the evolutionarily conserved C terminus (ECT1-11) (7). When we tested *ect* single mutants, we did not detect any significant immune phenotypes (*SI Appendix*, Fig. S2), potentially due to the functional redundancy of these ECTs. Indeed, when we subsequently generated high-order mutants based on their homology (7) through traditional crossing or CRISPR/Cas9, we found that both the *ect2/ect3* (*ect2/*3) double and the *ect2/ect3/ect4* (*ect2/3/4*) triple mutants from the YTHDFA clade showed a clear defect in PTI (Fig. 2*A*). Since these readers are known to be cytosolic localized (14, 25), their immune-deficient phenotype indicates that active recognition and processing of m^6^A mRNA by these proteins is necessary for PTI.

ECT2, ECT3, and ECT4 have been reported to function redundantly during plant development (8), and targets of ECT2 and ECT3 have significant overlap (14). To capture the ECT2-bound transcripts during PTI, we utilized the transgenic complementation plants *gECT2-YFP/ect2* to perform formaldehyde fixation and cross-linking immunoprecipitation-sequencing (FA-CLIP-Seq) with *35S:GFP* as a control under mock or elf18-treatment (Dataset S2). Consistent with previous findings (9), we found that ECT2 was mainly bound to translation stop site and 3′ UTRs, and elf18-treatment did not affect the overall pattern of the ECT2 binding (Fig. 2 *B* and *C*). Among the enriched peaks, UGUA is the most representative consensus sequence in the center of the peaks (Fig. 2*D*), as reported previously (9). Meanwhile, we compared the target genes identified under both mock and elf18-treatment. Only 131 transcripts were mock-specific, and 278 transcripts were elf18-specific, with the defense-related GO terms enriched in the pool of elf18-specific mRNAs (Fig. 2 *E* and *F*). However, most of the targets were bound by ECT2 under both conditions, consistent with the observations of the global m^6^A-seq.

To further investigate how reader proteins in the YTHDFA clade, ECT1-4, regulate the fate of mRNAs, we utilized a MS2-tethering system (26–28) to directly test the effects of ECT protein binding to target mRNAs (Fig. 2*G*). Protein of interest (POI), i.e., the ECT protein or the YFP control was fused to the C-terminal of the MS2 coat protein (MCP), whereas tandem MS2-binding sequences (MBS) were inserted into the 3′ UTR of the *Firefly Luciferase* (*FLUC*) mRNA where m^6^A modification and ECT2 binding were preferentially detected (Fig. 2 *B* and *C*). As an internal control, *Renilla Luciferase* (*RLUC*) was constitutively coexpressed. The resulting effectors and reporters were transiently coexpressed in *Nicotiana benthamiana* for 2 d. We found that ECT1 promoted the degradation of the *FLUC* mRNA with a similar level of reduction in the FLUC protein produced (Fig. 2 *H* and *I*), which is consistent with the recent finding on the *ect1* mutant in *Arabidopsis* (16). In contrast, tethering ECT3 to the *FLUC* mRNA enhanced the accumulation of the transcript with the corresponding increase in protein production (Fig. 2 *H* and *I*), indicating that ECT3-binding has the opposite effect on mRNA stability as ECT1. Distinct from ECT3, ECT2-binding increased the FLUC protein levels without significantly impacting the mRNA stability. These results indicate that in the *ect2/3* mutant, both the stability of the m^6^A-modified mRNAs and their translation were negatively affected. In this assay, we did not capture any detectable activity of ECT4. However, *ect2/3/4* has even a stronger development phenotype than *ect2/3* (8, 29) and a deficiency in PTI as *ect2/3* (Fig. 2*A*) suggesting ECT4 may have similar functions as ECT2 and ECT3. Therefore, we used the *ect2/3/4* mutant for further analyses.

### PTI-Inducible mRNAs Are Destabilized in an m^6^A-Dependent Manner.

**Fig. 3.**
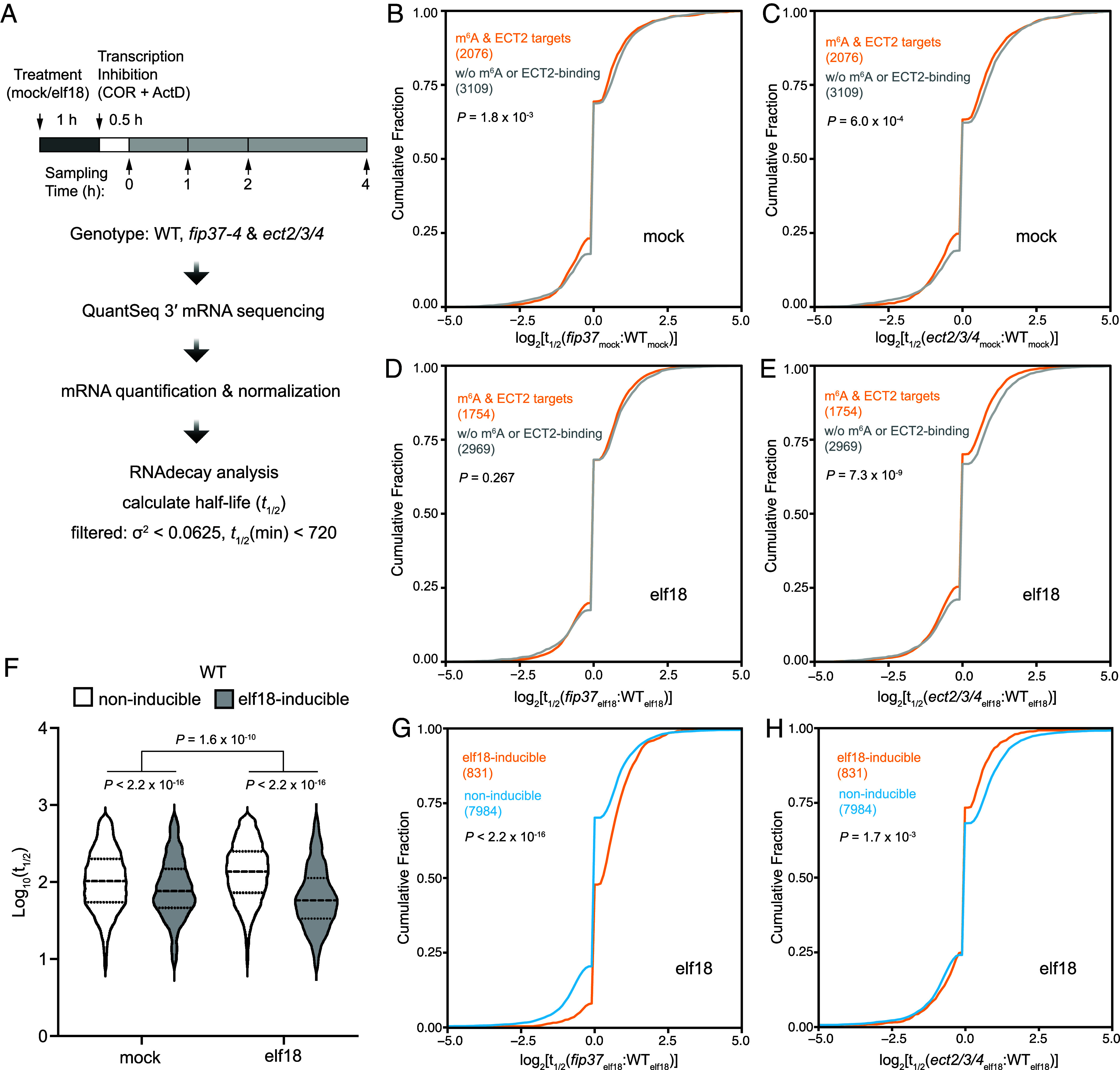
The elf18-induced mRNA decay is defective in the *fip37-4* mutant. (*A*) Schematic of mRNA decay assay for 10-d-old seedlings of WT, *fip37-4*, and *ect2/3/4* with mock- or elf18-treatment. (*B–E*) Cumulative distribution plots of mRNA half-life changes between WT and *fip37-4* (*B* and *D*) or *ect2/3/4* (*C* and *E*) under mock (*B* and *C*) or elf18 (*D* and *E*) condition. The plots compare m^6^A-modified ECT2-bound transcripts with those without (w/o) m^6^A modification or ECT2-binding. (*F*) Violin plot of half-lives of elf18-inducible or noninducible mRNAs in WT plants in response to mock- or elf18-treatment. (*G* and *H*) Cumulative distribution plots of mRNA half-life changes between WT and *fip37-4* (*G*) or *ect2/3/4* (*H*) in response to elf18-treatment. The plots differentiate between elf18-inducible and noninducible mRNAs. *P* values were calculated by the Mann–Whitney test (*B–H*) and two-way ANOVA (*F*).

To further elucidate the function of m^6^A during PTI, we performed global measurements of mRNA decay in WT, *fip37-4,* and *ect2/3/4* seedlings in response to mock or elf18 induction, followed by treatment of a cocktail of transcriptional inhibitors (cordycepin 150 µg/mL, actinomycin 10 µM) for 0.5 h prior to sample collection. The abundance of mRNA at Time 0 and at subsequent intervals of 1, 2, and 4 h was measured by QuantSeq (Fig. 3*A*). The decay rates of individual transcripts across different genotypes and conditions were determined using a previously reported mathematical modeling approach (30) (Dataset S3).

For comparison, we selected transcripts modified by m^6^A and bound by ECT2 (“m^6^A&ECT2 targets”) as the highly confident targets, and those without m^6^A or ECT2 binding (“w/o m^6^A or ECT2 binding”) as the control based on the m^6^A-IP-seq and FA-CLIP-seq data. Under mock condition, we observed that m^6^A-modified ECT2-targets were relatively less stable compared with nontargets in both *fip37-4* and *ect2/3/4* mutants, indicating m^6^A modification and ECT2/3 binding stabilize mRNA (Fig. 3 *B* and *C*). This observation aligns with previous findings suggesting that ECT2/3 can promote RNA stability (14, 15). Interestingly, under elf18-induced conditions, a similar result was only observed in *ect2/3/4*, not in the *fip37-4* mutant (Fig. 3 *D* and *E*), suggesting that during PTI, the m^6^A functions may not be fully determined by ECT2/3/4. Other reader proteins, such as ECT1 (16), which destabilizes mRNA (Fig. 2*H*) may also be involved.

Indeed, when we focused on transcripts that are transcriptionally induced by elf18 (“elf18-inducible”), we found, surprisingly, that they intrinsically had relatively shorter overall half-life compared to the noninducible ones under mock conditions, and the difference between the two groups became more pronounced upon elf18 treatment (Fig. 3*F*). This accelerated degradation observed in the elf18-inducible transcripts suggests the need for rapid turnover of these defense-related mRNAs, perhaps to avoid unnecessary growth inhibition (31). The role that m^6^A plays in this accelerated turnover of elf18-inducible mRNAs was further demonstrated by their enhanced relative stability in the writer complex mutant *fip37-4* compared to that in the WT background (Fig. 3*G*). Interestingly, this phenomenon was not observed in the *ect2/3/4* mutant (Fig. 3*H*), indicating that these three readers are not involved in the turnover of these elf18-inducible mRNAs. It is conceivable that other readers, such as ECT1, facilitate the RNA degradation during immune responses.

### ECT2/3/4 Are Required for Efficient Translation of m^6^A-Modified mRNAs during PTI.

The longer half-life of immune-inducible mRNAs in *fip37-4* (Fig. 3*G*) contradicts the PTI-deficient phenotype observed in this m^6^A writer mutant. The discrepancy suggests that this immune response must have additional layers of regulation by m^6^A modification. Besides mRNA stability, which does not seem to correlate with the mutant phenotype, we next considered the effect of m^6^A modification on translation because our MS2-tethering assays showed that ECT2 could enhance the translation efficiency of the target mRNA (Fig. 2 *H* and *I*) and a possible role for m^6^A modification in mediating translational activation during plant immune induction has not been previously examined. To fill this knowledge gap, we conducted polysome profiling on WT, *fip37-4*, and *ect2/3/4* seedlings exposed to either mock- or elf18-treatment for 1 h. Subsequently, we assessed translation efficiency (TE) by determining the ratio of mRNAs associated with polysomes to their total mRNA levels through QuantSeq (Dataset S4).

**Fig. 4.**
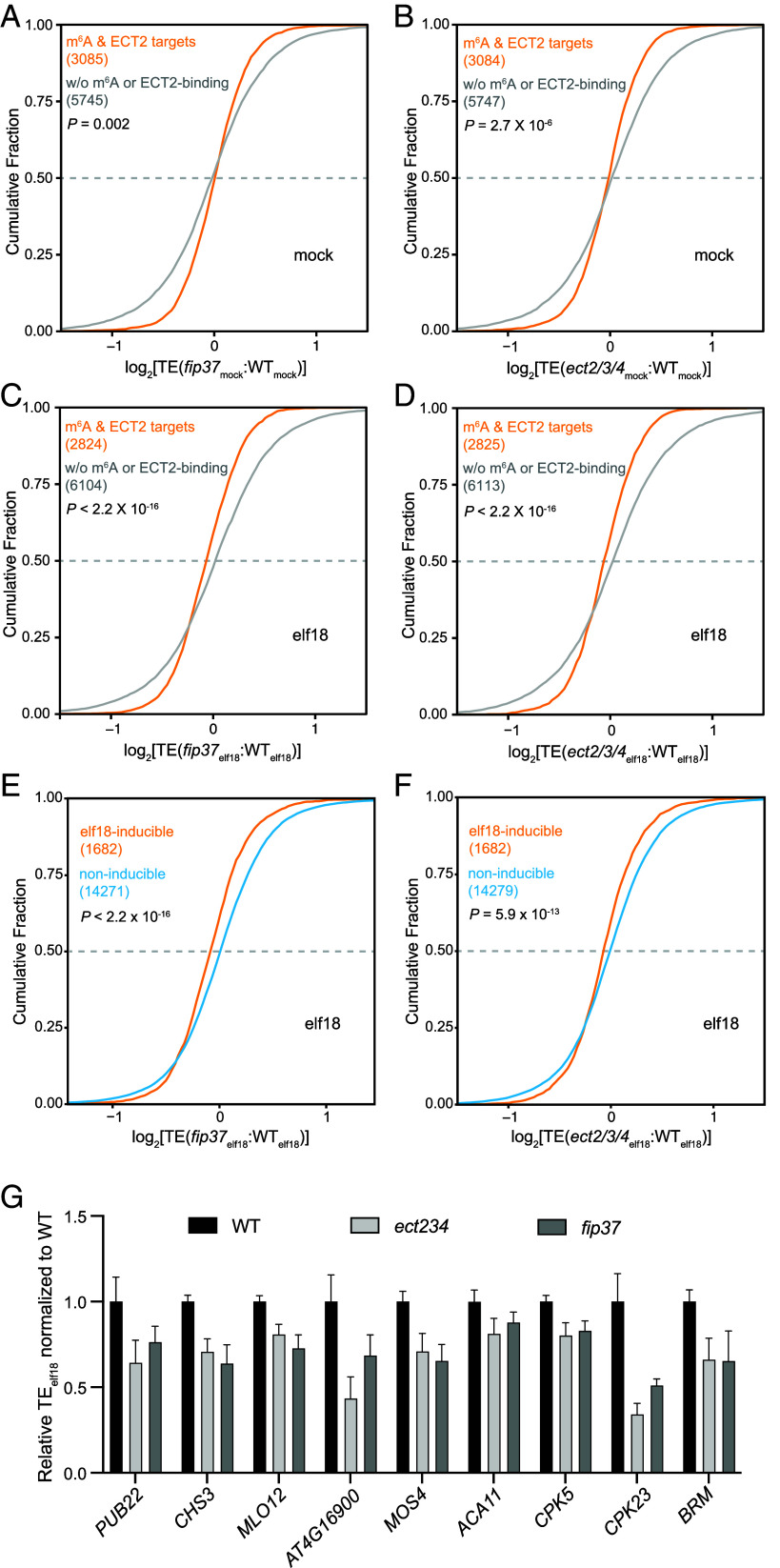
The m^6^A modification promotes translational efficiency (TE) of elf18-inducible transcripts through interactions with ECT2/3/4. (*A–D*) Cumulative distribution plots of TE changes between WT and *fip37-4* (*A* and *C*) or *ect2/3/4* (*B* and *D*) under mock (*A* and *B*) or elf18 (*C* and *D*) condition. The plots compare m^6^A-modified ECT2-bound transcripts and those without (w/o) m^6^A modification or ECT2-binding. (*E* and *F*) Cumulative distribution plots of TE changes between WT and *fip37-4* (*E*) or *ect2/3/4* (*F*) in response to elf18-treatment. The plots differentiate between elf18-inducible and noninducible mRNAs. *P* values were calculated by the Mann–Whitney test (*A–F*). (*G*) Relative TEs of representative immune-related mRNAs upon elf18-treatment in *fip37-4* and *ect2/3/4* mutants compared with WT. Error bars represent SD.

Calculations of TE revealed that, under mock treatment, WT plants and the *fip37-4* or *ect2/3/*4 mutants had no detectable overall TE difference (*SI Appendix*, Fig. S3*A*), whereas elf18 treatment led to a slight reduction in the overall TE in the mutants (*SI Appendix*, Fig. S3*B*). This reduction suggests a potential role for m^6^A and reader proteins in the efficient translation of defense-related transcripts during this immune response. For further analysis, we once again focused on comparisons between transcripts with m^6^A modification and ECT2-binding and transcripts with neither m^6^A modification nor ECT2-binding as the control. The cumulative curves, representing changes in TE between the mutants and WT, showed that m^6^A-modified ECT2-targets had lower TE values in the *ect2/3/4* mutant compared with the control transcripts under mock conditions, and a dramatic decrease in TE was observed in both *fip37-4* and *ect2/3/4* mutants upon elf18 treatment (Fig. 4 *A*–*D*). The only shared GO term enriched in the top 10% transcripts with the most impaired TE between *fip37-4* and *ect2/3/4* was “response to external stimulus” (*SI Appendix*, Fig. S4 *A* and *B*). This suggests that the translation of defense-related transcripts is defective without m^6^A modification or recognition. Indeed, while elf18-induced transcriptomes for WT and the mutants showed high levels of correlation (*SI Appendix*, Fig. S5), elf18-inducible transcripts were translated less efficiently in both *fip37-4* and *ect2/3/4* mutants upon elf18-treatment (Fig. 4 *E*–*G*). This indicates that the m^6^A writing and reading are crucial for rapidly reshaping of the transcriptome and translatome to facilitate the transient switch from growth to defense. The compromised translational response may underlie the observed deficiency in basal defense and PTI in the m^6^A writer and reader mutants.

**Fig. 5.**
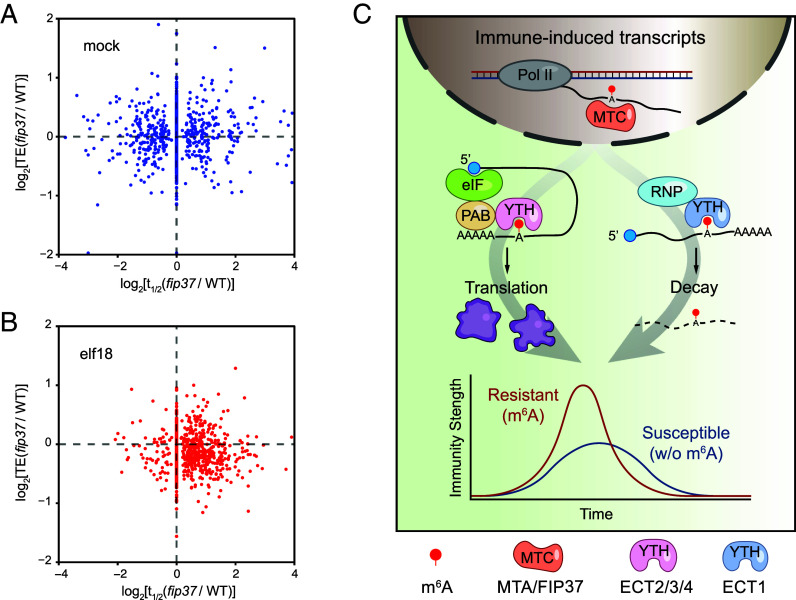
The m^6^A modification destabilizes immune-induced mRNAs while enhancing their translation efficiency (TE) upon elf18 induction. (*A* and *B*) Scatterplots of changes in elf18-inducible transcript stability (x axes) and TE (y axes) between *fip37-4* and WT under mock (*A*) or elf18 (*B*) condition. (*C*) Proposed model of m^6^A function during PTI. Upon elf18-treatment, immune-induced nascent transcripts are modified by the m^6^A methyltransferase complex (MTC). The modification destabilizes the transcripts through association with YTH domain-containing m^6^A readers proteins (YTH), such as ECT1, and RNA-binding proteins (RNP), while enhancing their TE by the activities of other YTH readers, such as ECT2/3/4, perhaps by recruiting eukaryotic translation initiation factors (eIF) and PAB protein. The resulting surge in the production of defense proteins leads to enhanced disease resistance.

To more clearly show how m^6^A-mediated mRNA stability and translation collectively shape the plant immune response, we made scatterplots to illustrate changes in RNA stability and TE between WT and the *fip37-4* mutant for the elf18-inducible transcripts. Comparing the elf18 treatment with mock, it is evident that the elf18-inducible transcripts exhibited extended half-lives in the m^6^A-deficient *fip37-4* (x axes in Fig. 5 *A* and *B*), yet showed lower TEs, compared to the WT (y axes in Fig. 5 *A* and *B*). These plots indicate that the dual function of m^6^A in PTI, destabilizing immune-induced mRNAs while promoting their TE, results in a pulsatile production of immune-associated proteins and enhanced disease resistance (Fig. 5*C*).

## Discussion

The m^6^A modification has been shown to modulate gene expression across eukaryotes (32). The expansion of the YTH protein family of m^6^A readers in plants suggests that m^6^A may play an even greater role in regulating plants’ response to environmental cues (7). However, the specific roles that the m^6^A regulatory network plays in plant defense against pathogen challenges remained ambiguous (16, 20, 21). Our resistance assays performed utilizing the *fip37-4* allele, known to cause m^6^A depletion in the transcriptome but remaining developmentally viable (5), revealed a positive role for FIP37-dependent m^6^A modification in both basal immunity (Fig. 1 *A* and *B*) and PTI (Fig. 1*D*). This is inconsistent with the result reported in a recent study in which the *mta* mutant of the catalytic component of the m^6^A writer complex was found to have enhanced basal resistance (21). It is possible that the severely retarded growth of the *mta* mutant led to stress responses which pleiotropically induced the immune response in the mutant without pathogen challenge, because when the *MTA* transcript was transiently knocked down in our *DEX*:*siMTA* lines, a deficiency in not only basal resistance, but also elf18-induced PTI was observed, similar to the *fip37-4* mutant plants (Fig. 1 *C*–*F*). Furthermore, higher-order m^6^A-reader mutants of the YTHDFA clade, *ect2/3* and *ect2/3/4,* are also partially compromised in PTI (Fig. 2*A*).

Though the primary focus of this study was on the function of m^6^A modification on mRNAs, this modification is also prevalent in various types of noncoding RNAs (ncRNAs). While m^6^A modification in ncRNAs might contribute to immune regulation, potentially through mechanisms such as chromatin remodeling (33, 34), the significant shifts in mRNA stability and translation efficiency combined with defective immune responses highlighted the critical role of m^6^A modification in mRNA for plant immunity.

The m^6^A-IP-seq and FA-CLIP-seq performed in this study allowed us to identify transcripts with elf18-specific m^6^A modifications and/or ECT2-binding. However, these transcripts constitute a relatively small proportion of the transcriptome. In future studies, quantitative measurements of m^6^A (35) can be implemented to more accurately define the dynamic changes in this modification during immune induction.

In comparison to the mammalian YTHDFs, which mainly function in promoting mRNA degradation and participating in translation (36), plants possess significantly more copies of YTHDF m^6^A readers, suggesting potentially more functional redundancy and diverse activities (7, 29). To discretely define the function of m^6^A readers, we employed the MS2-tethering system to assess their activities when bound to the *FLUC* reporter mRNA in planta. Our finding that the m^6^A reader ECT1 reduced the reporter mRNA levels and the production of the reporter protein (Fig. 2 *H* and *I*) is consistent with a recent publication demonstrating that ECT1 destabilized its targets through interaction with RNA binding proteins that mediate RNA degradation, such as DCP5, RH12, and TSN2 (16). Interestingly, overexpression of *ECT1* compromised plant immunity, and mutation of *ECT1* enhanced it (16), indicating the ECT1-mediated turnover of transcripts needs to be precisely controlled during immunity. In alignment with this hypothesis, stabilizing the defense-related transcripts in the RNA helicase mutant (*rh6/8/12*) led to autoimmunity and inhibition of growth (31). This suggests that m^6^A modification may facilitate the rapid degradation of immune-related mRNAs to prevent growth penalties, potentially through the action of decay-promoting readers, such as ECT1.

In contrast to ECT1, ECT2 and ECT3 enhanced protein production in the MS2-tethering assay (Fig. 2 *H* and *I*). In protein interactome studies, ECT2 and ECT3 were found to interact with poly(A)-binding (PAB) proteins via their N-terminal intrinsically disorder region (IDR) (15, 23, 37). PABs are known to promote both mRNA stability and translation by facilitating the looping of the 3′ poly(A) tail with the translation initiation complex at the 5′ cap (38). Interestingly, only upon elf18 treatment, the overall TE of the m^6^A-modified and ECT2-bound transcripts was impaired in both the *fip37-4* and *ect2/3/4* mutants (Fig. 4 *A*–*D*). This deficiency in translation was present in the elf18-inducible defense transcripts, suggesting that m^6^A modification and interaction with ECT2/3/4 are crucial to efficiently translate defense-related mRNAs.

In this study, we identified dual functions of m^6^A in regulating PTI in plants (Fig. 5*C*). It is well known that immune responses require rapid activation as well as swift deactivation; and m^6^A modification, which is deposited on the induced nascent mRNAs, supports these dynamics by enhancing translation and accelerating decay by the activities of different reader proteins. However, questions still remain: How do m^6^A readers compete with each other to dynamically control defense-related mRNA stability and translation? Whether and how do posttranslational modifications (39, 40) or phase separation (16, 41) affect the functions of ECT2/3/4 during the induction of PTI? With the framework established in this study, more precise studies will elucidate the different aspects of the m^6^A regulation of plant immune responses.

## Materials and Methods

### Plant Genotypes and Growth.

*Arabidopsis thaliana* and *Nicotiana benthamiana* were grown in soil under 12/12-h light/dark cycles at 22 °C with 55% relative humidity. All *Arabidopsis* lines used in this study are of the Columbia-0 accession. The *fip37-4* mutant (SALK_018636) and the *ect* single mutants were obtained from the *Arabidopsis* Biological Resource Center and listed in Dataset S5. The *gFIP37-GFP/fip37-4* transgenic line was provided by Hao Yu from the National University of Singapore (5). Previously published lines are *npr1-2* (42), *efr-1* (43), and *DEX:YFP* (44). The *ect2/3* mutant was generated by crossing *ect2* (SALK_002225C) with *ect3* (SALKseq_077502) and the *ect2/3/4* triple mutant was generated by CRISPR/Cas9 knock-out using a previous described method (45) with the guide RNA (gRNA) sequences shown in Dataset S5.

### Plasmid Construction and Plant Transformation.

To generate *DEX:siMTA*, primers P1/P2 (sequences for all primers used are listed in Dataset S6) were used to amplify the 332 bp fragment corresponding to the CDS of *MTA* to generate the PCR product 1 (*LIC1-MTA-LIC2*) with additional flanking sequences LIC1 and LIC2. PCR product 2 (*LIC4*-*LIC1-MTA-LIC2-LIC3*) was amplified by primers P3/P4 using *LIC1-MTA-LIC2* as a template to add another two flanking sequences LIC3 and LIC4. Both products containing the same sequence were inserted into pRNAi-LIC in the opposite orientation as described previously to generate the hairpin RNA (hpRNA) cassette (46). The hpRNA cassette was amplified using primers J1/J2 and ligated into pBAV154 via *Xbo*I/*Spe*I to build the construct pDEX:siMTA. The floral dip method was used to generate transgenic plants (47).

For generating the ECT CRISPR mutants, egg cell-specific CRISPR/Cas9 vector pHEE401E was used to obtain the *ect2/3/4* mutant (45). The website http://skl.scau.edu.cn/targetdesign/ was employed for designing gRNA targets and choosing the restriction enzymes for genotyping (Dataset S5). The CRISPR/Cas9 construct, pHEE401E-ect234, was transformed into WT plants using the floral dipping method. PCR and restriction enzyme digestion were applied to identify knock-out plants. The *ect2/3/4* mutant plants identified in the T3 generation were confirmed by Sanger-sequencing of gRNA-targeted regions. In the T2 and T3 generations, Cas9-F and Cas9-R primers were used to identify Cas9-free plants, and Cas9-free T4 plants were also confirmed through viable growth screening on MS plates supplemented with 20 mg/L hygromycin B.

For complementation of the mutation in ECT2, the ECT2 coding sequence was inserted into the plasmid pLIC-YFP by ligation-independent cloning (LIC) (48) to obtain *35S:ECT2-YFP*. To construct *gECT2-YFP,* the *35S* promoter was replaced by the *ECT2* native promoter through restriction cloning *Eco*RI. The construct was transformed to the *ect2* plants for complementation. For the MS2 assay, the pMCP-LIC vector was generated by replacing YFP sequence in pYFP-LIC with the HA-MCP sequence. CDS sequences of YFP, ECT1, ECT2, ECT3, or ECT4 were amplified and inserted into the pMCP-LIC vector by ligation-independent cloning. Six tandem MS2 binding sites were amplified from pEGAD-M6 (27) and inserted into the pFLUC-RLUC vector (22) after the stop codon of *FLUC* to generate the *pFLUC-M6-RLUC* reporter.

### *Hpa* Noco2 Infection Assay.

*Hpa* Noco2 infection assay was performed as previously described (49). For *Hpa* Noco2 infection, 12-d-old plants were grown under 12/12-h light/dark cycles. The plants were sprayed with a suspension of 3-5 × 10^4^ spores/mL in H_2_O and covered with a dome to achieve 100% saturated humidity for 1 d. The plants were then exposed to ambient humidity for 3 d before being covered again. 7 d after the initial infections, spores were collected by suspending infected plants in 5 mL of water. Spores were then counted in a hemacytometer under a microscope.

### Bacterial Infection Assay.

The basal resistance assay was performed as previously described (49). Leaves from 3.5-wk-old plants were infiltrated with *Psm* ES4326 (OD_600nm_ = 0.0001) suspended in 10 mM MgCl_2_. Bacterial growth was scored on Day 0 and Day 3 by serial dilutions in 10 mM MgCl_2_. For the *DEX:siMTA* silencing lines, 50 µM dexamethasone (DEX) or mock (H_2_O) was sprayed on leaves 1 d prior to infiltration with *Psm* ES4326 (OD_600nm_ = 0.0001). The elf18-induced protection assay was performed as previously described (22). Leaves from 3.5-wk-old plants were infiltrated with 1 µM elf18 in H_2_O or mock (H_2_O) or pre-treated for 1 d with 50 µM DEX or mock (H_2_O) prior to 1 µM elf18 treatment. After 1 d, the same leaves were then infiltrated with *Psm* ES4326 (OD_600nm_ = 0.001) and bacterial growth was scored 2 d later.

### LC-MS/MS.

*Arabidopsis* plants were grown on MS plates (1/2 MS basal salts, 1% sucrose, and 0.8% agar) for 6 d. Plants were then transferred to 6-well plates containing liquid MS media (1/2 MS basal salts, 1% sucrose) and grown for four additional days. For elf18 treatment, the growth media were replaced with fresh liquid MS media with or without 1 µM elf18 and incubated for 1 h. The sample was collected, immediately frozen in liquid nitrogen, ground using the Genogrinder (SPEX SamplePrep), and subjected to total RNA isolation using the Direct-zol RNA Miniprep Plus Kit (Zymo). mRNA was enriched twice through poly(A) selection using Oligo d(T)_25_ Magnetic Beads (NEB). Around 200 ng purified mRNA was digested in a two-step manner with nuclease P1 (1 μL, Sigma-Aldrich) in 20 μL reaction buffer containing 10 mM of NH_4_OAc (pH= 5.3) at 42 °C for 2 h. Then, 1 μL of shrimp alkaline phosphatase (rSAP, NEB) was added along with 2.5 μL of 10× CutSmart buffer (NEB) and incubated at 37 °C for 2 h. After the incubation, the sample was diluted with additional 35 μL water and filtered with 0.22 μm filters (4 mm diameter, Millipore) and 6 to 8 μL of the entire solution was injected as one replicate into C18 reverse phase column coupled to Agilent 6460 LC-MS/MS spectrometer in positive electrospray ionization mode. The nucleosides were quantified by using retention time and the nucleoside to base ion mass transitions (282-to-150). For all the quantification, a mock control with only digestion buffers and enzymes was included each time and was later used for the subtraction of baseline signals. Quantification was performed in comparison with the standard curve, obtained from pure nucleoside standards running with the same batch of samples. The m^6^A level was calculated as the ratio of m^6^A to A.

### m^6^A Immunoprecipitation and Sequencing.

3.5-wk-old WT plants were infiltrated with 10 µM elf18 or mock (H_2_O). Whole leaf tissue (~1.5 g per replicate, three replicates per treatment) was collected and flash frozen in liquid nitrogen 1 h after infiltration. Total RNA was then extracted using TRIZOL (Ambion) according to the manufacturer’s instructions. mRNA was isolated from the total RNA using the Dynabeads mRNA DIRECT purification kit (ThermoFisher). mRNA concentration was adjusted to 15 ng/µL in 100 µL and fragmented using a Bioruptor ultrasonicator (Diagenode) with 30 cycles of 30 s on/off. m^6^A-immunoprecipitation (m^6^A-IP) and library preparation were performed according to the published protocol (50). Specifically, the total input mRNA used was reduced to 1 µg. Correspondingly, 2.5 µg of anti-m^6^A antibody was used. Input and m^6^A-IP eluted RNA libraries were constructed using the TruSeq Stranded mRNA kit (Illumina) following the standard protocol. Sequencing was carried out using Illumina HiSeq 4000 according to the manufacturer’s instructions.

### Data Processing for m^6^A Sequencing.

Three replicates of m^6^A input and IP libraries were aligned to the *Arabidopsis* genome (TAIR10) using Bowtie2.0 (51). Reads were assigned to genes by alignment to the exons of the longest isoform of each gene. For m^6^A peak calling, a sliding window of 100 nt in length with a step of 50 nt was used to determine read coverage of the longest isoform of each gene in each library. Read coverage in each window was pseudocount transformed and normalized by the median window read coverage. In m^6^A IP libraries, windows with a greater than 3 peak-over-median (POM) score were condensed. These regions were normalized by the read coverage in the corresponding input library to determine a peak-over-input (POI) score. A region of greater than 3 POI in at least two of the three replicates was determined to be a putative m^6^A methylation site. Putative peaks are considered specific to a condition if none of the windows in the peak is found with a significant POI score in the other condition.

### FA-CLIP-seq.

FA-CLIP-seq was modified from the previous study (9). *gECT2-YFP* and *35S:YFP* plants were grown on MS plates for 6 d. Plants were then transferred to 6-well plates containing liquid MS media and grown for four additional days. For elf18 treatment, the growth media were replaced with fresh liquid MS media with or without 1 µM elf18 and incubated for 1 h. For input, plant tissues were collected right after treatment. For immunoprecipitation samples, plants were fixed and crosslinked by 1% formaldehyde under a vacuum to enhance infiltration. Two replicates were collected for the experiment. The lysate from 2 g tissues was partially digested with RNase T1 and immunoprecipitated by 25 µL GFP-trap magnetic beads to pull down ECT2-bound RNA fragments. The other steps were same as described in the previous study (9). The final libraries were sequenced using the Illumina NovaSeq 6000 platform. The method of peak calling was similar to that used for m^6^A peak calling. ECT2 binding sites were determined by a sliding window analysis of read density between IP and input samples for each genotype and condition. Briefly, for each transcript model reads were assigned to windows along the length of the transcript model with a sliding window step size of 10 nt. Read density was compared for each window in the IP and input sample to calculate the POI score (IP/input). Sensitivity of the POI was determined by analyzing the YFP samples and set to the 90th percentile of POI scores across YFP libraries, with all windows below this POI score removed from further analysis. For each transcript model, significance of the POI score was determined by Z-score analysis. Windows with a Z-score below 2.576 were removed from future analysis. The false discovery rate (FDR) was calculated by calling peaks with input and IP samples inverted (input/IP). Windows with a POI Z-score of >2.576 and an FDR < 0.05 were considered putative ECT2 binding sites.

### ECT-MS2 Tethering Assay.

*Agrobacteria* were cultured overnight, spun down, and resuspended in the infiltration buffer (10 mM MgCl_2_, 10 mM MES pH = 5.6, and 200 µM acetosyringone) for 2 h and diluted to OD_600nm_ = 0.8, and strain harboring pFLUC-M6-RLUC was mixed with that of pMCP-YFP or pMCP-ECT in a 1:3 ratio and infiltrated into *N. benthamiana* leaves. Samples were collected for RNA and protein extraction after 2-d expression. Protein samples were subject to dual-luciferase assay as previously described (17), whereas RNA samples were extracted by TRIZOL according to the manufacturer’s instructions and followed by cDNA synthesis and qPCR. All qPCR primers used are described in Dataset S6.

### RNA Degradation Assay.

WT, *fip37-4,* and *ect2/3/4* plants were grown on MS plates for 6 d. Plants (6 to 10 seedlings) were then transferred to 12-well plates containing liquid MS media and grown for four additional days. For elf18 treatment, the growth media were replaced with fresh liquid MS media with or without 1 µM elf18 and incubated for 1 h. At half an hour after adding inhibitors of mRNA transcription (0.6 mM cordycepin, 10 µM actinomycin), plants were collected (time 0) and flash frozen in liquid nitrogen. The following timepoints were subsequently collected 1, 2, and 4 h later. All frozen tissue samples were processed with TRIZOL to isolate total RNA according to the manufacturer’s instruction. n = 3.

### Polysome Profiling Assay.

WT, *fip37-4*, and *ect2/3/4* plants were grown on MS plates for 6 d. Plants were then transferred to 6-well plates containing liquid MS media and grown for four additional days. For elf18 treatment, the growth media were replaced with fresh liquid MS media with or without 1 µM elf18 and incubated for 1 h. Polysome profiling was performed as previously described (22). Briefly, 0.3 g tissues for each sample were ground in liquid nitrogen and extracted with 1.2 mL polysome extraction buffer [200 mM Tris pH 9.0, 200 mM KCl, 35 mM MgCl_2_, 25 mM EGTA, 5 mM DTT, 1 mM phenylmethanesulfonyl fluoride, 50 µg mL^−1^ cyclohexamide, 50 µg mL^−1^ chloramphenicol, 1% (v/v) Brij-35, 1% (v/v) Igepal CA630, 1% (v/v) Triton X-100, 1% sodium deoxycholate, and 1% (v/v) polyoxyethylene 10 tridecyl ether]. The resulting lysate (0.8 mL) was loaded on a sucrose gradient (15 to 60%) and centrifuged at 4 °C for 10 h (35,000 rpm; Ti-SW41 rotor), and 0.1 mL of lysate was saved as input. Polysome profiles and fractions were collected using a fractionator and 254 nm UV monitor. Fractions corresponding to polysomes were pooled, as determined by their sedimentation patterns. RNAs were isolated from input and polysome pooled fractions by TRIZOL-LS according to the manufacturer’s instructions. n = 3.

### QuantSeq for RNA Decay and Translation Efficiency.

Total RNA (500 ng) was used for library preparation via the QuantSeq 3′ mRNA Seq Library Prep FWD Kit from Illumina (Lexogen). Sequencing was performed at the Duke Center for Genomic and Computational Biology using NovaSeq 6000 or NovaSeq X for 50 bp single-end reads. Raw reads were trimmed to remove adaptors and poly(A) sequences by using Trim Galore (52), and then mapped to the *Arabidopsis* genome TAIR10 by using the STAR RNA sequencing aligner (53). The raw counts for each gene were calculated by HTSeq and normalized by DESeq2 for further analyses (54).

To calculate the RNA decay rate, the Bioconductor RNAdecay package was utilized to normalize the data, model mRNA decay, and compare genotype effects. With limitation of the program, different genotypes with the same treatment were fitted to the decay models together. For each transcript, the best decay model was selected by the lowest Akaike information criterion (AICc), and the initial decay rate α and the decay of decay rate β of the transcript were estimated by that model. Half-life of mRNA was calculated as t_1/2_ = ln(2)/α. For the downstream analysis, the transcripts with the variance σ^2^ of the model less than 0.0625 and half-life less than 720 min across all genotypes under the same condition were selected.

To measure the translation efficiency (TE), TE for each transcript was calculated in individual replicates as mRNA(polysome)/mRNA(input), and the average TE from all three replicates was used for global analysis. DESeq2 was used for differential expression analysis, and the inducible transcripts of WT were detected by fold-change of mRNA(input) >1.5 and an adjusted *P*-value < 0.05.

## Supplementary Material

Appendix 01 (PDF)

Dataset S01 (XLSX)

Dataset S02 (XLSX)

Dataset S03 (XLSX)

Dataset S04 (XLSX)

Dataset S05 (XLSX)

Dataset S06 (XLSX)

## Data Availability

The sequencing data are available through the National Center for Biotechnology Information under accession number PRJNA1118749 (55).
