# Supplementary material for: m6A modification plays an integral role in mRNA stability and translation during pattern-triggered immunity
Source: Proc Natl Acad Sci U S A. 2024 Aug 8;121(33):e2411100121. doi: 10.1073/pnas.2411100121 (PMC11331096; doi:10.1073/pnas.2411100121)
Supplement: Supplementary file 1 — Appendix 01 (PDF) [file pnas.2411100121.sapp.pdf]

Supporting Information for

m^6A modification plays an integral role in mRNA stability and translation during pattern-triggered immunity

Tianyuan Chen[1*], George H. Greene[1*], Jonathan Motley[1*], Musoki Mwimba[1], Guan-Zheng Luo[2], Guoyong Xu[1,a], Sargis Karapetyan[1], Yezi Xiang[1], Chang Liu[2], Chuan He[2], and Xinnian Dong[1**]

Dr. Xinnian Dong

Email: xdong@duke.edu

This PDF file includes:

Figures S1 to S5

Other supporting materials for this manuscript include the following:

Dataset S1 to S6

SI Appendix

Figures

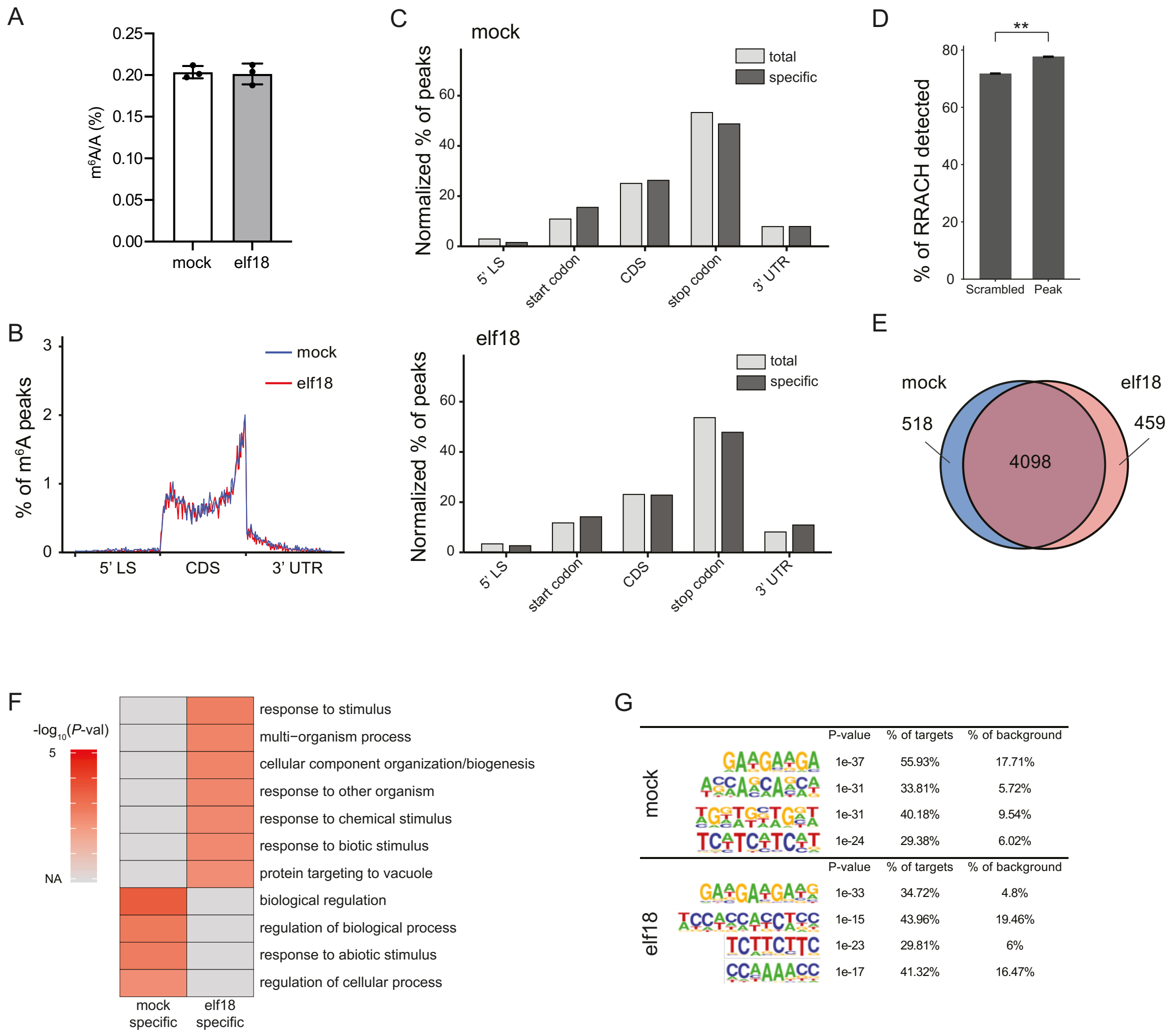

Fig. S1. Global analyses of m^{6}A modification in response to elf18 treatment. (*A*) Quantification of global m^{6}A modification with and without elf18 induction using liquid chromatography-tandem mass spectrometry. The m^{6}A level was calculated as the ratio of m^{6}A to A. (*B*) Global distribution of m^{6}A modification in transcripts under mock- or elf18-treated condition (percentage to total). Position of m^{6}A modification in the 5' leader sequence (5'LS), the coding sequence (CDS), and

the 3’ untranslated region (UTR) of mRNA was normalized for each region. (*C*) Distribution of m^6A modification in 5’LS, start codon ± 50 nt (start), CDS, stop codon ± 50 nt (stop), and 3’UTR. Upper panel, mock total and mock-specific (specific) m^6A modification distribution; lower panel, elf18 total and elf18-specific m^6A modification distribution. (*D*) FIMO (Find Individual Motif Occurrences) analysis of all m^6A modifications for the presence of the RRACH-consensus compared to scrambled background sequences with equivalent length and nucleotide composition of modification site sequences. chi-squared test, ** $P < 0.01$. (*E*) Venn diagram of overlap between m^6A modified mRNAs in mock- and elf18-treated samples. (*F*) Heatmap of Gene Ontology enriched terms for the m^6A-modified mRNAs present under mock (mock-specific) or elf18 (elf18-specific) condition. (*G*) Enrichment of *de novo* consensus in regions 150 nt flanking either end of the m^6A modification site. Top panel, consensus in mock-specific transcripts. Bottom panel, consensus in elf18-specific transcripts. Consensus enrichment performed with HOMER against a randomized background of equivalent length and nucleotide composition.

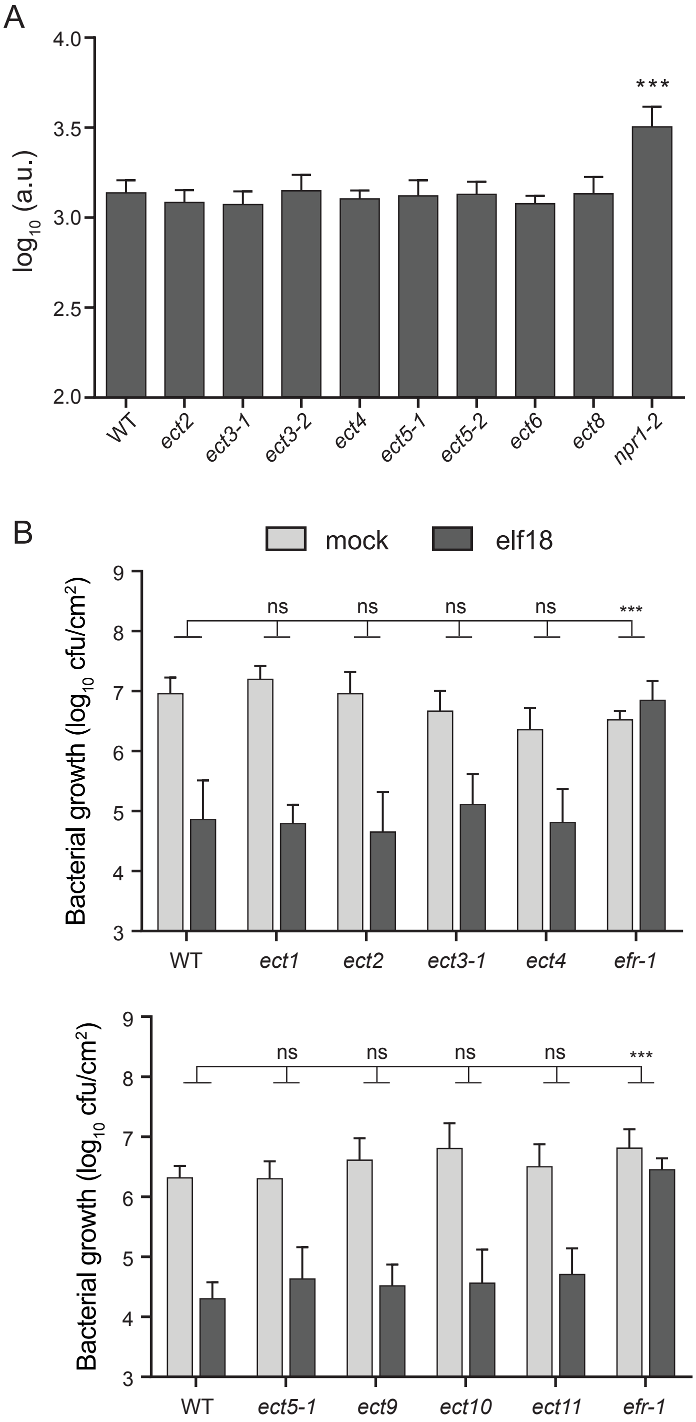

Fig. S2. Immune phenotypes of the m^{6}A reader single mutants. (*A*) Basal resistance to a bacterial pathogen. Leaves from 3.5-week-old plants were infiltrated with *Pseudomonas syringae* pv *tomato* DC3000-LUX (*Pst-LUX*) (OD_{600nm} = 0.0001). Bacterial growth was

measured by luminescence after 3 days ($n = 6$). *npr1*, *nonexpressor of pathogenesis-related 1* mutant known to have enhanced disease susceptibility. (*B*) elf18-induced resistance to a bacterial pathogen. Mature leaves were infiltrated with 1 μM elf18 or mock (H_2O). After 1 day, the same leaves were infiltrated with *Psm* ES4326 ($OD_{600nm} = 0.001$) and bacterial growth was scored 2 days later ($n = 8$). *efr-1*, an elf18-receptor mutant. All error bars represent 95% confidence intervals. Data were analyzed by the Student's t-test (*A*) and two-way ANOVA (*B*) with Bonferroni post hoc test. ***, $P < 0.001$; ns, not significant.

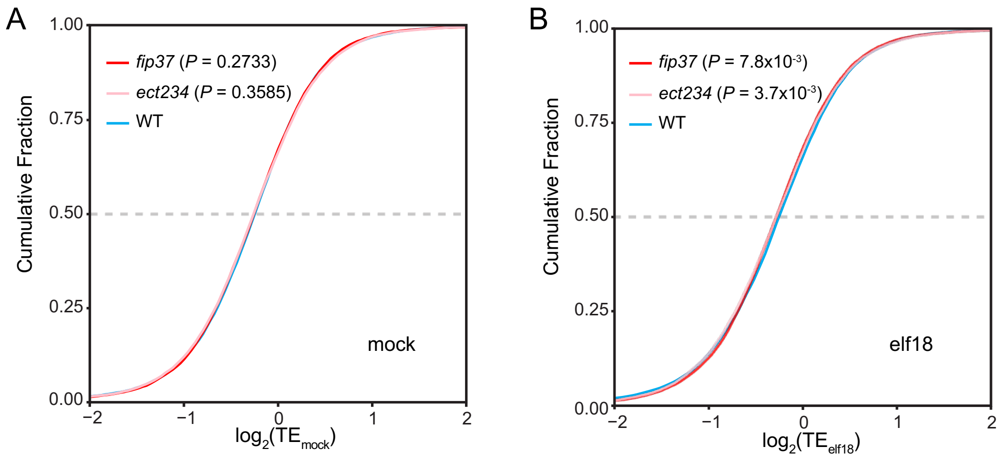

Fig. S3. Global translation efficiency (TE) in the m^{6}A writer and reader mutants. (*A* and *B*) Cumulative distribution of TE in WT, *fip37-4*, and *ect2/3/4* plants under mock (*A*) or elf18 (*B*) condition. *P* values were calculated by the Mann-Whitney test.

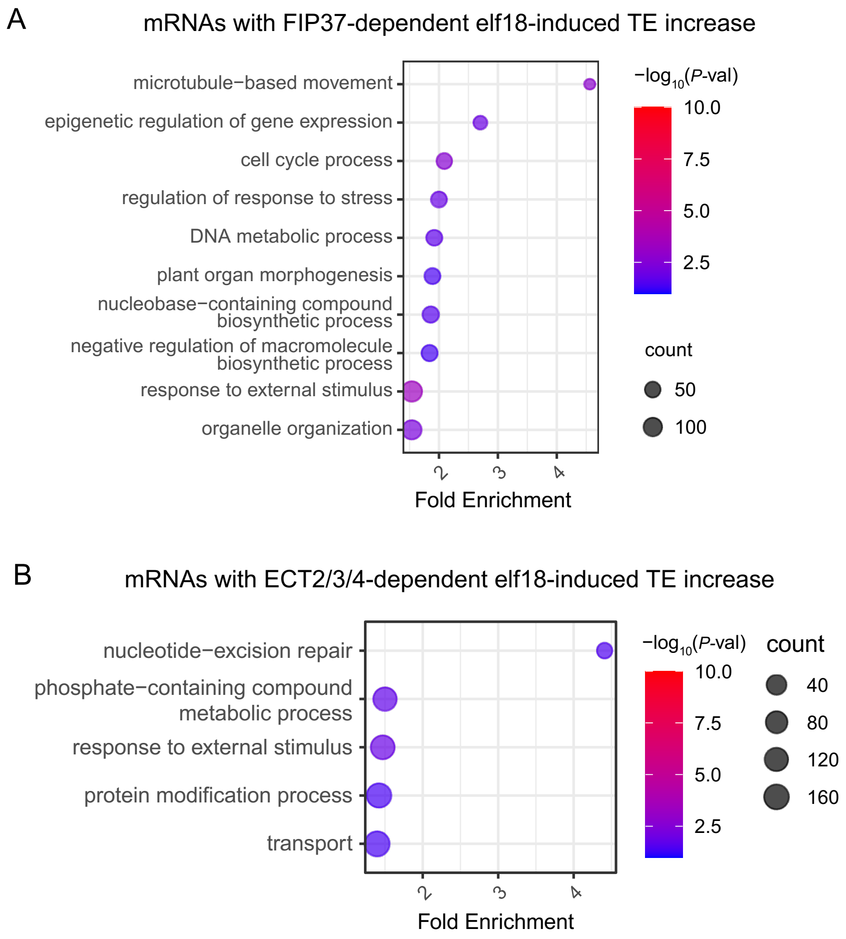

Fig. S4. Gene Ontology term enrichment for transcripts whose elf18-induced TE are dependent on FIP37 and ECT2/3/4. The 10% most affected mRNAs in *fip37-4* (*A*) or *ect2/3/4* (*B*) compared to WT were analyzed.

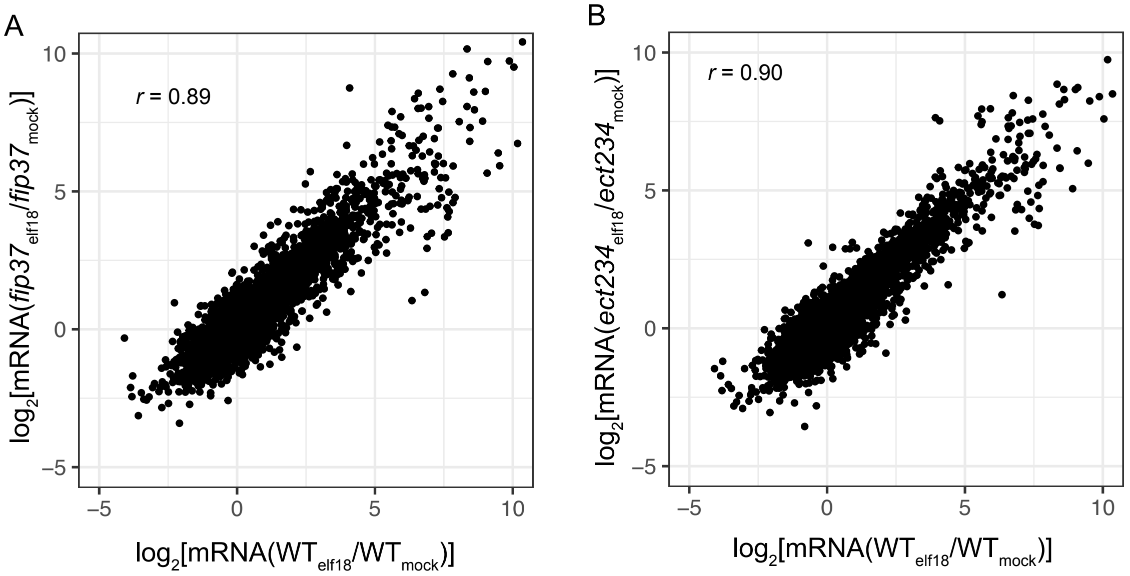

Fig. S5. The transcriptional changes induced upon elf18 treatment were not defective in the m^6A mutants. The scatterplots illustrate the correlation of transcriptional induction by elf18 treatment between WT and *fip37-4* (*A*) or *ect2/3/4* (*B*) mutant.

Datasets (separate files)

Dataset S1. m^{6}A peaks under mock- and elf18-treatments

Dataset S2. ECT2-binding peaks under mock- and elf18-treatments

Dataset S3. RNA decay rates under mock- and elf18-treatments

Dataset S4. Translation efficiency under mock- and elf18-treatments

Dataset S5. *ect* mutants used in this study

Dataset S6. Primers used in this study
